# Chronic disease clusters and health-related quality of life among individuals with musculoskeletal pain: a Northern Finland Birth Cohort 1966 study

**DOI:** 10.1093/fampra/cmaf057

**Published:** 2025-07-17

**Authors:** Eveliina Heikkala, Jaro Karppinen

**Affiliations:** Research Unit of Health Sciences and Technology, University of Oulu, Oulu, Finland; Medical Research Center Oulu, University of Oulu and Oulu University Hospital, Oulu, Finland; Wellbeing Services County of Lapland, Rovaniemi, Finland; Research Unit of Health Sciences and Technology, University of Oulu, Oulu, Finland; Medical Research Center Oulu, University of Oulu and Oulu University Hospital, Oulu, Finland; Rehabilitation Services of Wellbeing Services County of South Karelia, Lappeenranta, Finland

**Keywords:** musculoskeletal pain, chronic disease clusters, latent class analysis, multimorbidity, health-related quality of life

## Abstract

**Background:**

Musculoskeletal (MSK) pain is known to influence health-related quality of life (HRQoL), but the role of co-occurring chronic diseases in HRQoL in a MSK pain population has been less studied. This study aimed to evaluate (i) whether chronic disease clusters are related to HRQoL and (ii) whether these relationships differ in magnitude from those between the number of chronic diseases and HRQoL among people with MSK pain.

**Material and methods:**

The Northern Finland Birth Cohort 1966 and its 46-year data collection point were used. The chronic disease clusters for individuals reporting any MSK pain within the past year were previously formulated using latent class analysis and consisted of: *Psychiatric* (co-existing mental health disorder, substance use disorder, and asthma), *Metabolic* (referring to the burden of metabolic diseases), and *Relatively healthy*. HRQoL was measured with a 15-dimension questionnaire. General linear regression model was used.

**Results:**

Among 4490 participants, both the *Psychiatric* and *Metabolic* clusters associated with clinically significantly reduced HRQoL, when contrasted with the *Relatively healthy* cluster, but the association was stronger for the *Psychiatric* cluster. Similarly, the adjusted mean difference in HRQoL was higher for the *Psychiatric* cluster than for the multimorbidity (two or more diseases) category when compared with the reference categories (*Relatively healthy* cluster and no chronic diseases, respectively).

**Conclusions:**

The present findings imply the clinical relevance of the previously identified chronic disease clusters and suggest that pure counts of chronic diseases may not be enough to describe the role of chronic diseases in HRQoL in MSK pain.

**Key messages**

- Mental and metabolic health are likely important to HRQoL in MSK pain.
- Pure counts of chronic diseases may not be enough to describe their role in HRQoL.
- The clinical relevance of the chronic disease clusters at population level is highlighted.

## Introduction

An essential aim of our healthcare systems and governments is to improve and maintain individuals’ health-related quality of life (HRQoL), as reduced HRQoL correlates, for instance, with higher healthcare use [1, 2] and mortality risk [2, 3]. Importantly, as a shift away from a reduced HRQoL may be difficult to enact [4–6], emphasis should be placed on the early identification of elements resulting in such reductions, as well as targeted preventive actions. Various instruments have been developed to capture the wide spectrum of the individual-level psychosocial and physical elements that construct HRQoL. One of these is a validated 15-dimension (15D) questionnaire based on 15 questions designed to evaluate HRQoL from these physical and mental perspectives [7–9].

Musculoskeletal (MSK) pain is among the top diseases to which reduced HRQoL can be attributed [10, 11], with the measurement of such pain including the years lived with disability based on estimates from the World Health Organization Burden of Diseases Database [12]. However, not all people with MSK pain experience long-term reductions in HRQoL, since for individuals with acute MSK pain (e.g. low back pain), symptoms are usually alleviated over time [13], and a segment of people with chronic MSK conditions, such as osteoarthritis, are able to maintain their functional capacity and sufficient HRQoL [5, 6]. Still, there is a subgroup of individuals with MSK pain whose HRQoL remains worse [5, 6].

The existing literature has identified several factors associated with reduced HRQoL in pain populations. These include, for example, female sex [5, 14], somatic symptom burden [15], depressive and anxiety symptoms [6, 16], certain lifestyle factors [4, 6, 17] and pain-related factors, such as duration [16] and distress/social stress [14, 16]. Unsurprisingly, several chronic diseases also contribute negatively to HRQoL among people with MSK pain [5, 6, 18, 19], but very limited research has been devoted to the role of specific patterns of chronic diseases in HRQoL, and the one study that does exist in this vein focused only on osteoarthritis [19]. Given that multimorbidity (i.e. having more than one chronic disease [20]), is highly common, affecting 12.9% to 95.1% of patients in primary care [21], focusing on single diseases or only their counts may obscure the development of validated conclusions about the subgroups with MSK pain that are most vulnerable to reduced HRQoL. Identification of individuals with the most unfavorable profile of chronic diseases in relation to the HRQoL is of importance to provide evidence for trials on MSK pain management.

In our prior study, we identified three chronic disease clusters (*Psychiatric*, *Metabolic*, and *Relatively healthy*) in a sample with MSK pain from a population-level birth cohort and found them to be differently associated with severe MSK pain [22]. This study aims to disentangle the clinical relevance of these chronic disease clusters by studying whether the clusters are also differently associated with reduced HRQoL in midlife. As a secondary aim, we compared descriptively the strength of these associations to those of the number of chronic diseases and HRQoL. Further exploration of the clinical relevance of the identified clusters is warranted, particularly by evaluating HRQoL as an outcome, since pain severity reflects only one aspect of the total burden and subjective suffering. We hypothesized that belonging to both the *Psychiatric* (characterized by the co-occurrence of mental health problems, substance use problems, and asthma) and *Metabolic* (characterized by the accumulation of obesity, hypertension, and diabetes) clusters relates to reduced HRQoL, relative to the *Relatively healthy* cluster. We also hypothesized that both *Psychiatric* and *Metabolic* clusters have stronger association with reduced HRQoL than the number of chronic diseases themselves.

## Material and methods

### The Northern Finland Birth Cohort 1966 (NFBC1966)

**Figure 1.**
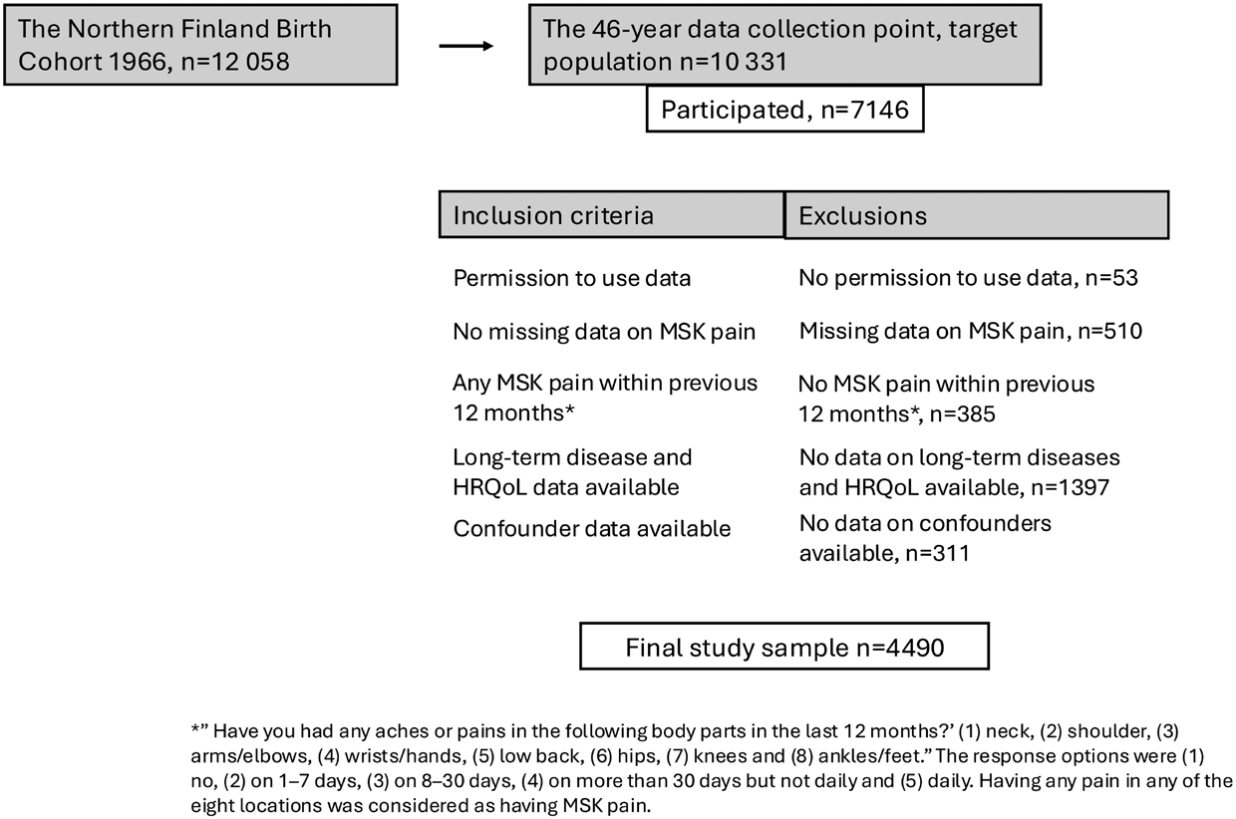
Flowchart of the study sample

The NFBC1966 is a large birth cohort of Northern Finns born in 1966 in Oulu and Lapland, the northernmost provinces of Finland (with 12058 live-born children [23]). This cross-sectional study concentrates on the 46-year data collection point of the cohort, which started in 2012. Of the target population of 10331 participants, 7146 comprised the dataset for this study based on their participation in the data collection [24]. The selection procedure for the final study sample is presented in detail in Fig. 1. In short, the inclusion criteria were reporting any MSK pain within the previous 12 months; providing self-reported data on chronic diseases, HRQoL, and potential confounders; and providing written permission to use their data in the research. The study was approved by the Ethics Committee of the Northern Finland Hospital District 94/2011 (12.12.2011) and followed the Declaration of Helsinki.

### Health-related quality of life (HRQoL)

HRQoL was defined by the 15D questionnaire, which contains 15 dimensions (scaled 0–5, with 0 = no problems and 5 = extreme problems/inability) related to the current health state. These include breathing, mental function, speech (communication), vision, mobility, usual activities, vitality, hearing, eating, elimination, sleeping, distress, discomfort and symptoms, sexual activity, and depression. The dimensions can be used separately or as a single index score measure, which is referred to as a 15D score. The 15D score is calculated by summing up the set of population-based utility-of-preference weighted points of the 15 dimensions, leading to a final number ranging from 0 to 1 (0 = dead, 1 = full HRQoL) [8]. When assessed separately, each dimension is also scaled in a similar way. The minimal important change (MIC) in the 15D score (i.e. an improvement or reduction in the score and thus in experienced HRQoL) has been estimated at 0.015 [25]. A difference of 0.035 or more can be considered as much improved or much reduced HRQoL [25]. The 15D questionnaire is a standardized tool that has been shown to be valid in both general and pain populations [7, 9]. In this study, the 15D score and its dimensions were assessed as continuous variables.

### Chronic disease clusters

We used previously formed chronic disease clusters, labeled as *Psychiatric*, *Metabolic*, and *Relatively Healthy*, as the exposure in the analyses [22]. There clusters were identified using the latent class cluster analysis (LCA), which is a statistical method designed to divide heterogeneous populations into homogeneous groups (“clusters”) in terms of studied variables, i.e. here, chronic diseases. LCA assumes that there exists an uncovered, latent variable that explains the co-occurrence of studied variables within each cluster and categorizes individuals into the most probable cluster, based on the posterior cluster membership probabilities.

The precise formulation and characteristics of these latent class clusters are presented elsewhere [22]. In short, the *Psychiatric* cluster was characterized by the co-occurrence of a diagnosed mental health disorder, substance use disorder, and asthma; the *Metabolic* cluster was defined by accumulated metabolic diseases, such as obesity, hypertension, and diabetes; and the *Relatively Healthy* cluster was indicative of a low probability of any included chronic disease. These clusters were formulated using latent class cluster analysis, and selection of the most appropriate model was based on fit indices [22]. The *Relatively Healthy* cluster was utilized as the reference category in the present analyses. Chronic diseases apart from self-reported obesity (body mass index 30 kg/m2 or over) were identified through self-reported physician diagnoses (yes/no) and chosen for inclusion based on their longevity, non-traumatic and non-infectious background, and non-pain-inducing clinical characteristics. The included chronic diseases are shown in Supplementary Table 1.

To evaluate the secondary aim, we trichotomized the sum of chronic diseases into zero, one, and two or more chronic diseases. In these analyses, zero chronic diseases was employed as the reference category.

To estimate current mental health symptoms in addition to diagnoses, we calculated the score points for the Hopkins Symptom Checklist-25 (HSCL-25 questionnaire) and dichotomized them into 1.55 or over (“severe mental distress”) versus under 1.55 (“mild mental distress”) [26].

### Potential confounders

Sex (female/male), smoking (current smoker/former smoker/non-smoker [22]) and educational level (primary = 9 years or less, secondary = 10–12 years, and tertiary = more than 12 years) were included in the study as potential confounders, as they were each deemed to fulfill the criteria for a confounder [4,14,22] while not functioning as collinears or mediators. All these data, except for those pertaining to sex, which were based on birth records, were gathered from the 46-year questionnaire.

### Statistical analysis

The distribution of the included variables was presented as percentages and numbers or means and standard deviations (SDs) according to the chronic disease clusters and for the total sample, as appropriate. The Kruskal–Wallis test and chi-square test were used for the continuous and categorical variables, respectively. Mean for each dimension of 15D was calculated for the clusters and visualized using a spider chart. The associations between the chronic disease clusters and HRQoL were measured with general linear regression models, and described by the beta (ß) coefficient and 95% confidence intervals (CIs), using the *Relatively healthy* cluster as the reference. ß coefficients and their 95% CIs were presented as unadjusted and adjusted for sex, educational level, and smoking (fully adjusted). A general linear regression model, presented as unadjusted and fully adjusted, was also employed to study the associations between the number of chronic diseases and HRQoL, using the “zero chronic diseases” category as the reference. ß coefficients characterized the mean difference in the 15D score between the studied exposure categories and the reference category. The statistical significance was set at 0.05. All analyses were conducted using a complete case approach with SPSS, version 27.0.

## Results

The study population included 4490 participants, of whom 57% were females (Table 1). Most had a secondary-level education (65%), and nearly half of the participants were non-smokers (48%). The mean of HRQoL was 0.925 (SD: 0.066) on a score scale of 0 to 1. A total of 10% of the participants belonged to the *Metabolic* cluster, 3% to the *Psychiatric* cluster, and the rest (87%) to the *Relatively healthy* cluster.

**Table 1.** Characteristics of the individuals reporting musculoskeletal pain at 46 years according to latent class clusters of chronic diseases, presented as % (n) if otherwise indicated.

	Metabolic (*n* = 466)	Psychiatric (*n* = 128)	Relatively healthy (*n* = 3896)	Total (*n* = 4490)	*P* value
Sex					<.001
Males	51 (238)	41 (53)	42 (1636)	43 (1927)	
Females	49 (228)	59 (75)	58 (2260)	57 (2563)	
Educational level					
Primary	13 (62)	16 (20)	6 (218)	7 (300)	<.001
Secondary	69 (319)	61 (78)	65 (2536)	65 (2933)	
Tertiary	18 (85)	23 (30)	29 (1142)	28 (1257)	
Smoking					<.001
Non-smoker	36 (168)	31 (39)	50 (1944)	48 (2151)	
Former smoker	34 (159)	24 (31)	27 (1045)	27 (1235)	
Current smoker	30 (139)	45 (58)	23 (907)	25 (1104)	
Number of chronic diseases					<.001
Zero	0	0	52 (2008)	45 (2008)	
One	0	0	35 (1376)	31 (1376)	
Two or more	100 (466)	100 (128)	13 (512)	24 (1106)	
HSCL-25^*^					<.001
Mild mental distress	71 (312)	36 (43)	81 (3000)	79 (3355)	
Severe mental distress	29 (126)	64 (75)	19 (681)	21 (882)	
Health-related quality of life					
Mean (SD)	0.892 (0.084)	0.815 (0.103)	0.932 (0.057)	0.925 (0.066)	<.001

^*^Hopkings Symptom Checklist-25 for depressive and anxiety symptoms; frequencies vary due to missing data.

*P* value was calculated by chi-square test for categorical variables and by Kruskal-Wallis test for continuous variables.

SD = standard deviation.

**Figure 2.**
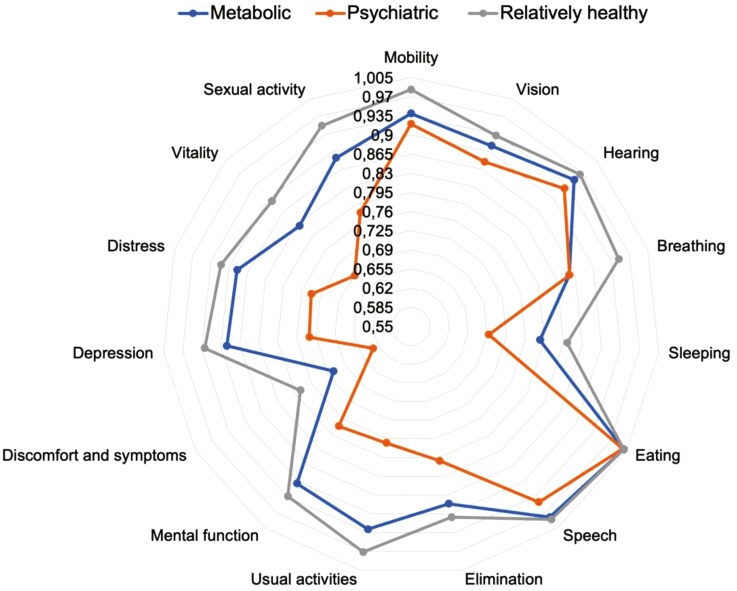
A spider chart of the mean values of the 15 dimensions according to the latent class clusters of chronic diseases. Division lines are visualized by every 0.035-point increase in the mean.

In relation to the *Relatively healthy* cluster, the *Metabolic* cluster included more males than females (51% vs. 49%), and both the *Metabolic* and *Psychiatric* clusters had a higher prevalence of participants with primary education only (13% and 16% vs 6%) and participants who currently smoked (30% and 45% vs 23%, respectively). A total of 64% of the *Psychiatric* cluster participants had severe mental distress symptoms when estimated using HSCL-25. The *Relatively healthy* cluster had a higher mean HRQoL than the total study sample, while the means were much lower in the *Metabolic* (0.892, SD: 0.084) and *Psychiatric* (0.815, SD:0.103) clusters. The *Relatively healthy* cluster also had the highest mean values in nearly every dimension of the 15D, while the opposite was observed for the *Psychiatric* cluster (Fig. 2).

Table 2 shows the associations between the chronic disease clusters and HRQoL. In the unadjusted models, both the *Metabolic* and *Psychiatric* clusters were found to be associated with reduced HRQoL when compared with the *Relatively healthy* cluster (ß −0.040, 95% CI −0.046 to −0.0324; ß −0.117, 95% CI −0.128 to −0.106, respectively). In the models adjusted for sex, educational level, and smoking, the strength of these associations remained practically the same (ß -0.038, 95% CI −0.044 to −0.032; ß −0.112, 95% CI −0.123 to −0.101, respectively). Both adjusted ß values exceeded the MIC value of the 15D tool used for measuring HRQoL.

**Table 2.** Associations between health-related quality of life and latent class clusters of chronic diseases among individuals reporting any musculoskeletal pain (*n* = 4490).

	*Unadjusted*			*Adjusted* ^*^		
	ß	95% CI	*P* value	ß	95% CI	*P* value
*Metabolic*	**−0.040**	**−**0.046 to **−**0.034	<.001	**−0.038**	**−**0.044 to **−**0.032	<.001
*Psychiatric*	**−0.117**	**−**0.128 to **−**0.106	<.001	**−0.112**	**−**0.123 to **−**0.101	<.001
*Relatively healthy*	Reference			Reference		

^*^Adjusted for sex, educational level, and smoking.

Bolded values are statistically significant.

ß=beta coefficient.

CI = confidence interval.

Table 3 represents the associations between the number of chronic diseases and HRQoL. Both categories (one and two or more chronic diseases) were associated with reduced HRQoL, when using zero chronic diseases as the reference category, but the association of two or more chronic diseases was clearly stronger than that of one chronic disease (adjusted ß −0.053, 95% CI −0.057 to −0.048 for two or more chronic diseases; adjusted ß −0.018, 95% CI −0.022 to −0.014 for one chronic disease).

**Table 3.** Associations between health-related quality of life and number of chronic diseases among individuals reporting any musculoskeletal pain (*n* = 4490).

	*Unadjusted*			*Adjusted* ^*^		
	ß	95% CI	*P* value	ß	95% CI	*P* value
*Two or more*	**−0.056**	**−**0.060 to **−**0.051	<.001	**−0.053**	**−**0.057 to **−**0.048	<.001
* One*	**−0.019**	**−**0.024 to **−**0.015	<.001	**−0.018**	**−**0.022 to **−**0.014	<.001
* Zero*	Reference			Reference		

^*^Adjusted for sex, educational level, and smoking.

Bolded values are statistically significant.

ß = beta coefficient.

CI = confidence interval.

## Discussion

This population-based study on a large birth cohort was designed to determine whether distinct chronic disease clusters are differently associated with a reduction in HRQoL when studying people with MSK pain. Belonging to the *Psychiatric* cluster, characterized by the co-occurrence of diagnosed mental health disorder, substance use disorder, and asthma, was associated with highly reduced HRQoL, when compared with the *Relatively healthy* people. Moreover, the *Metabolic* cluster, including participants with metabolic diseases, was also found to have a relationship with reduced HRQoL, but this difference was of a lower magnitude than that of the *Psychiatric* cluster and HRQoL. While focusing only on the count of diseases, people with one or two or more chronic diseases had a lower mean of HRQoL when contrasted to people with no chronic diseases.

Altogether, the present findings indicate that both mental and metabolic health are related to reduced HRQoL among individuals with MSK pain. Importantly, the observed mean differences in the 15D between the *Psychiatric* and *Metabolic* clusters, and the *Relatively healthy* cluster exceeded not only the minimal important difference level of 0.015 but also the level of a markedly changed status (0.035) [25]. These findings supplement the existing knowledge of the role of accumulated chronic diseases in HRQoL from the general population [27] by considering the pain population in particular and establishing the clinical relevance of these previously identified chronic disease clusters.

The relationship of co-occurring MSK pain and mental health disorders (*Psychiatric* cluster) with HRQoL was, however, substantially stronger than that between the *Metabolic* cluster and HRQoL. Indirectly, it also seemed that there might be differences in the HRQoL between *Psychiatric* and *Metabolic* clusters, given that the adjusted CIs did not overlap. Psychiatric morbidity and symptoms do not only themselves strongly relate to reduced HRQoL [6, 10, 16], but they also seem to exacerbate the inverse associations between physical conditions and HRQoL [28, 29], which are findings that also support the present study’s results. Nearly two-thirds of the participants in the *Psychiatric* cluster had severe symptoms of depression and anxiety, as estimated by the HSCL-25, which suggests that most of those individuals were in unbalanced treatment situations in terms of mental health disorders. The pain phenotype, which was for nearly half of the participants in the *Psychiatric* cluster severe, i.e. prolonged, bothersome, and multisite [22], may, to some extent, explain the present results related to this particular group [30–32]. However, as pain symptoms were a priori considered as potential mediators rather than confounders, they were not controlled for in analyses. Here, the lowest single dimension values were seen in discomfort and symptoms (which also included pain/ache), sleeping, and vitality dimensions of HRQoL. It should be noted that the dyad of mental health disorders and poor sleep is already well recognized in pain research [33, 34]. However, nearly all other dimensions but those related to psychiatric diseases were at the lowest level in the *Psychiatric* cluster as well, emphasizing the comprehensive impairment of HRQoL in this cluster.

It is important not to neglect those people without the burden of mental health disorders but who have instead been diagnosed with metabolic diseases. Particularly, as the prevalence of the *Metabolic* cluster (10%) was three-fold relative to that of the *Psychiatric* cluster (3%), and as these people have a heightened risk for cardiovascular diseases and mortality [35]. Obesity may also expose one to worsening pain [36], and thus, the maintenance of reduced HRQoL [30, 37]. In a study of patients with osteoarthritis, increased body mass index was observed to be weakly associated with poorer HRQoL, but it was not found to predict worsening of HRQoL over a 3-year follow-up period [38]. Interestingly, concurrent cardiovascular disease did not modify these cross-sectional relationships, thereby placing indirectly emphasizing the presence of obesity and other metabolic diseases in the *Metabolic* cluster, given that 87% of these participants had obesity, and 84 % had hypertension.

Both the *Psychiatric* and *Metabolic* clusters included participants with all having at least two chronic diseases, while the *Relatively healthy* cluster had a wider spectrum when it came to the number of chronic diseases, and only 13% had multimorbidity. Therefore, chronic disease clusters can be considered a reflection of multimorbidity patterns in this study population [20]. When evaluating only the number of diseases, having two or more chronic diseases was associated with a notable reduction in HRQoL (−0.053), but this value did not exceed the mean difference between the *Psychiatric* and *Relatively healthy* clusters (−0.112) and was actually close to that of the *Metabolic* and *Relatively healthy* clusters (−0.038). This is particularly interesting, given that 48% of the individuals in the *Relatively healthy* cluster had at least one chronic disease. These results imply that the presence of multimorbidity, irrespective of the knowledge of the distribution of specific diseases, is related to reduced HRQoL, but most importantly, if multimorbidity includes mental health and substance use burden, HRQoL seems to be lower. Additionally, these findings can be viewed as suggestive of the need for a deviation from pure counts to the clustering of chronic diseases in MSK pain research.

Prior longitudinal studies have convergently shown that multimorbidity predicts reduced HRQoL among patients with osteoarthritis [5, 6, 19]. Similar findings were observed in a cross-sectional study in which individuals with chronic low back pain were examined [39]. In a study by Zhao et al. [19], a combination of other musculoskeletal diseases with cardiovascular disease had the greatest impact on HRQoL reduction, while others [5, 6, 39] focused only on the number of diseases or used certain indices, such as the Charlson Comorbidity Index. These results by Zhao et al. [19] may not be fully comparable to the present ones, given certain limitations, like the fact that they did not include any diseases from the mental health spectrum. Overall, the present study is the first to disentangle the role of naturally clustered, non-primarily pain-causing chronic diseases in HRQoL in a general population with MSK pain.

The definite strength of this study is contingent upon the large population-based study sample of Northern Finns. The NFBC1966 originally covered nearly all live-born children born in Northern Finland (around half of Finland from a geographical viewpoint). Even though the long follow-up of the cohort led to some attrition, trending slightly toward males and people from lower socioeconomic status that is typical in survey designs [40, 41], 69% of the original cohort members participated in the full 46-year data collection point [24]. Moreover, while the study sample used here exhibited some differences in background variables in comparison to those who participated but who were excluded due to, for example, missing data [42], these were substantially minor.

The following limitations require some attention. Due to the cross-sectional design, the determination of no reverse causality cannot be excluded, and any conclusions about cause-and-effect relationships are not valid. Also, the study is based on self-reported data, which may be vulnerable to desirability and recall biases, thereby leading to inaccuracies in the measurement tools. At the same time, it should be noted that HRQoL, which was the outcome of this study, may be difficult to objectively measure. Finally, there was some sparsity in the chronic disease data in terms of the treatment balance of chronic diseases, except for mental disorders. Thus, further clinical studies are warranted.

## Conclusions

The co-occurrence of mental health disorders, substance use disorders, and asthma alongside the accumulation of obesity, hypertension, and diabetes were associated with clinically meaningfully reduced HRQoL. Of these relationships, the first seemed to be especially strong and even stronger than the association between multimorbidity and HRQoL. The present findings imply the clinical relevance of the previously identified chronic disease clusters and therefore suggest that individuals who have mental health or metabolic burdens may be in greater need of more intensive and multi-professional cooperative treatment and rehabilitation measures to maintain or improve their HRQoL. The results also slightly suggest that pure counts of chronic diseases may not be enough to describe the role of chronic diseases in HRQoL in MSK pain. We encourage future studies to evaluate whether achieving a sufficient treatment balance of accumulated chronic diseases (e.g. diabetes and hypertension), compared to not achieving such a balance, is an element that influences these detected associations. Moreover, further studies with longitudinal HRQoL data are needed.

## Supplementary Material

cmaf057_suppl_Supplementary_Tables_1

## Data Availability

NFBC data are available from the University of Oulu, Infrastructure for Population Studies. Permission to use the data can be applied for research purposes via an electronic material request portal. In the use of data, we follow the EU General Data Protection Regulation (679/2016) and the Finnish Data Protection Act. The use of personal data is based on a cohort participant’s written informed consent in their latest follow-up study, which may cause limitations to its use. Please, contact the NFBC project center (NFBCprojectcenter(at)oulu.fi) and visit the cohort website (www.oulu.fi/nfbc) for more information.

## References

[1] Killingmo RM , Chiarotto A, van der Windt DA, et al Modifiable prognostic factors of high costs related to healthcare utilization among older people seeking primary care due to back pain: an identification and replication study. BMC Health Serv Res 2022;22:793. https://doi.org/10.1186/s12913-022-08180-2 35717179 PMC9206382

[2] Singh JA , Nelson DB, Nichol KL. Recent health-related quality of life, but not change, predicted mortality and healthcare utilization. J Clin Epidemiol 2021;140:13–21. https://doi.org/10.1016/j.jclinepi.2021.08.023 34433010

[3] Phyo AZZ , Freak-Poli R, Craig H, et al Quality of life and mortality in the general population: a systematic review and meta-analysis. BMC Public Health 2020;20:1596. https://doi.org/10.1186/s12889-020-09639-9 33153441 PMC7646076

[4] Arvidsson S , Arvidsson B, Fridlund B, et al Health predicting factors in a general population over an eight-year period in subjects with and without chronic musculoskeletal pain. Health Qual Life Outcomes 2008;6:98. https://doi.org/10.1186/1477-7525-6-98 19014459 PMC2636776

[5] Costa D , Lopes DG, Cruz EB, et al Trajectories of physical function and quality of life in people with osteoarthritis: results from a 10-year population-based cohort. BMC Public Health 2023;23:1407. https://doi.org/10.1186/s12889-023-16167-9 37480019 PMC10362599

[6] Han A , Gellhorn AC. Trajectories of quality of life and associated risk factors in patients with knee osteoarthritis: findings from the osteoarthritis initiative. Am J Phys Med Rehabil 2018;97:620–7. https://doi.org/10.1097/PHM.0000000000000926 29547449

[7] Sintonen H. The 15D-measure of health-related quality of life. II. Feasibility, reliability and validity of its health state descriptive system. National Centre for Health Program Evaluation, Working Paper 41, Melbourne, 1994.

[8] Sintonen H. The 15D measure of health-related quality of life. II. Feasibility, reliability and validity of its valuation system. National Centre for Health Program Evaluation, Working Paper 42, Melbourne, 1995.

[9] Vartiainen P , Mäntyselkä P, Heiskanen T, et al Validation of EQ-5D and 15D in the assessment of health related quality of life in chronic pain. Pain 2017;158:1577–85. https://doi.org/10.1097/j.pain.0000000000000954 28715354

[10] Agborsangaya CB , Lau D, Lahtinen M, et al Health-related quality of life and healthcare utilization in multimorbidity: results of a cross-sectional survey. Qual Life Res 2013;22:791–9. https://doi.org/10.1007/s11136-012-0214-7 22684529

[11] González-Chica DA , Hill CL, Gill TK, et al Individual diseases or clustering of health conditions? Association between multiple chronic diseases and health-related quality of life in adults. Health Qual Life Outcomes 2017;15:244. https://doi.org/10.1186/s12955-017-0806-6 29268792 PMC5740772

[12] Sebbag E , Felten R, Sagez F, et al The world-wide burden of musculoskeletal diseases: a systematic analysis of the World Health Organization Burden of Diseases Database. Ann Rheum Dis 2019;78:844–8. https://doi.org/10.1136/annrheumdis-2019-215142 30987966

[13] Wallwork SB , Braithwaite FA, O’Keeffe M, et al The clinical course of acute, subacute and persistent low back pain: a systematic review and meta-analysis. CMAJ 2024;196:E29–46. https://doi.org/10.1503/cmaj.230542 38253366 PMC10805138

[14] Vartiainen P , Heiskanen T, Sintonen H, et al Health-related quality of life and burden of disease in chronic pain measured with the 15D instrument. Pain 2016;157:2269–76. https://doi.org/10.1097/j.pain.0000000000000641 27355183

[15] Fujii T , Oka H, Katsuhira J, et al Association between somatic symptom burden and health-related quality of life in people with chronic low back pain. PLoS One 2018;13:e0193208. https://doi.org/10.1371/journal.pone.0193208 29462181 PMC5819824

[16] Keeley P , Creed F, Tomenson B, et al Psychosocial predictors of health-related quality of life and health service utilisation in people with chronic low back pain. Pain 2008;135:142–50. https://doi.org/10.1016/j.pain.2007.05.015 17611036

[17] Lopes DG , Costa D, Cruz EB, et al Association of physical activity with physical function and quality of life in people with hip and knee osteoarthritis: longitudinal analysis of a population-based cohort. Arthritis Res Ther 2023;25:14. https://doi.org/10.1186/s13075-023-02996-x 36703210 PMC9878813

[18] Bair MJ , Wu J, Damush TM, et al Association of depression and anxiety alone and in combination with chronic musculoskeletal pain in primary care patients. Psychosom Med 2008;70:890–7. https://doi.org/10.1097/PSY.0b013e318185c510 18799425 PMC2902727

[19] Zhao T , Winzenberg T, Aitken D, et al The impact of comorbidities on health-related quality of life of people with osteoarthritis over 10 years. Rheumatology (Oxford) 2021;61:139–45. https://doi.org/10.1093/rheumatology/keab358 33871587

[20] Johnston MC , Crilly M, Black C, et al Defining and measuring multimorbidity: a systematic review of systematic reviews. Eur J Public Health 2019;29:182–9. https://doi.org/10.1093/eurpub/cky098 29878097

[21] Violan C , Foguet-Boreu Q, Flores-Mateo G, et al Prevalence, determinants and patterns of multimorbidity in primary care: a systematic review of observational studies. PLoS One 2014;9:e102149. https://doi.org/10.1371/journal.pone.0102149 25048354 PMC4105594

[22] Heikkala E , Oura P, Paananen M, et al Chronic disease clusters are associated with prolonged, bothersome, and multisite musculoskeletal pain: a population-based study on Northern Finns. Ann Med 2023;55:592–602. https://doi.org/10.1080/07853890.2023.2177723 36773018 PMC9930817

[23] University of Oulu. Northern Finland Birth Cohort 1966. Oulu: University of Oulu. Available at: http://urn.fi/urn:nbn:fi:att:bc1e5408-980e-4a62-b899-43bec3755243

[24] Nordström T , Miettunen J, Auvinen J, et al Cohort Profile: 46 years of follow-up of the Northern Finland Birth Cohort 1966 (NFBC1966). Int J Epidemiol 2022;50:1786–7j. https://doi.org/10.1093/ije/dyab109 34999878 PMC8743124

[25] Alanne S , Roine RP, Räsänen P, et al Estimating the minimum important change in the 15D scores. Qual Life Res 2015;24:599–606. https://doi.org/10.1007/s11136-014-0787-4 25145637

[26] Veijola J , Jokelainen J, Läksy K, et al The Hopkins Symptom Checklist-25 in screening DSM-III-R axis-I disorders. Nord J Psychiatry 2003;57:119–23. https://doi.org/10.1080/08039480310000941 12745774

[27] Kanesarajah J , Waller M, Whitty JA, et al Multimorbidity and quality of life at mid-life: A systematic review of general population studies. Maturitas 2018;109:53–62. https://doi.org/10.1016/j.maturitas.2017.12.004 29452782

[28] Pan T , Anindya K, Devlin N, et al The impact of depression and physical multimorbidity on health-related quality of life in China: a national longitudinal quantile regression study. Sci Rep 2022;12:21620. https://doi.org/10.1038/s41598-022-25092-7 36517510 PMC9750988

[29] Van Wilder L , Devleesschauwer B, Clays E, et al The impact of multimorbidity patterns on health-related quality of life in the general population: results of the Belgian Health Interview Survey. Qual Life Res 2022;31:551–65. https://doi.org/10.1007/s11136-021-02951-w 34424487 PMC8847309

[30] Heikkala E , Paananen M, Merikanto I, et al Eveningness intensifies the association between musculoskeletal pain and health-related quality of life: a Northern Finland Birth Cohort Study 1966. Pain 2022;163:2154–61. https://doi.org/10.1097/j.pain.0000000000002609 35135992 PMC9578528

[31] Nicholl BI , Macfarlane GJ, Davies KA, et al Premorbid psychosocial factors are associated with poor health-related quality of life in subjects with new onset of chronic widespread pain - results from the EPIFUND study. Pain 2009;141:119–26. https://doi.org/10.1016/j.pain.2008.10.022 19059720 PMC2631192

[32] Rundell SD , Patel KV, Krook MA, et al Multi-site pain is associated with long-term patient-reported outcomes in older adults with persistent back pain. Pain Med 2019;20:1898–906. https://doi.org/10.1093/pm/pny270 30615144

[33] Bilterys T , Siffain C, De Maeyer I, et al Associates of insomnia in people with chronic spinal pain: a systematic review and meta-analysis. J Clin Med 2021;10:3175. https://doi.org/10.3390/jcm10143175 34300341 PMC8304652

[34] Karimi R , Mallah N, Scherer R, et al Sleep quality as a mediator of the relation between depression and chronic pain: a systematic review and meta-analysis. Br J Anaesth 2023;130:747–62. https://doi.org/10.1016/j.bja.2023.02.036 37059623

[35] Afshin A , Forouzanfar MH, Reitsma MB, et al; GBD 2015 Obesity Collaborators. Health effects of overweight and obesity in 195 countries over 25 years. N Engl J Med 2017;377:13–27. https://doi.org/10.1056/NEJMoa1614362 28604169 PMC5477817

[36] Tanguay-Sabourin C , Fillingim M, Guglietti GV, et al; PREVENT-AD Research Group. A prognostic risk score for development and spread of chronic pain. Nat Med 2023;29:1821–31. https://doi.org/10.1038/s41591-023-02430-4 37414898 PMC10353938

[37] Mutubuki EN , Beljon Y, Maas ET, et al The longitudinal relationships between pain severity and disability versus health-related quality of life and costs among chronic low back pain patients. Qual Life Res 2020;29:275–87. https://doi.org/10.1007/s11136-019-02302-w 31531837 PMC6962124

[38] Renaudin L , Guillemin F, Pouchot J, et al The presence of cardiovascular disease does not modify the weak impact obesity has on health-related quality of life in patients with hip osteoarthritis in the KHOALA cohort. Joint Bone Spine 2018;85:233–8. https://doi.org/10.1016/j.jbspin.2017.02.006 28242173

[39] Santos H , Henriques AR, Branco J, et al Health-related quality of life among spondyloarthritis and chronic low back pain patients: results from a nationwide population-based survey. Qual Life Res 2023;32:383–99. https://doi.org/10.1007/s11136-022-03274-0 36308590

[40] Mindell JS , Giampaoli S, Goesswald A, et al; HES Response Rate Group. Sample selection, recruitment and participation rates in health examination surveys in Europe--experience from seven national surveys. BMC Med Res Methodol 2015;15:78. https://doi.org/10.1186/s12874-015-0072-4 26438235 PMC4595185

[41] Tolonen H , Helakorpi S, Talala K, et al 25-year trends and socio-demographic differences in response rates: Finnish adult health behaviour survey. Eur J Epidemiol 2006;21:409–15. https://doi.org/10.1007/s10654-006-9019-8 16804763

[42] Heikkala E , Oura P, Ho E, et al Accumulation of long-term diseases is associated with musculoskeletal pain dimensions among middle-aged individuals with musculoskeletal pain. Eur J Pain2023a;27:438–48. https://doi.org/10.1002/ejp.2070 36560860

