# Supplementary material for: Chronic disease clusters and health-related quality of life among individuals with musculoskeletal pain: a Northern Finland Birth Cohort 1966 study
Source: Fam Pract. 2025 Jul 17;42(4):cmaf057. doi: 10.1093/fampra/cmaf057 (PMC12268870; doi:10.1093/fampra/cmaf057)
Supplement: cmaf057_suppl_Supplementary_Tables_1 [file cmaf057_suppl_supplementary_tables_1.pdf]

Supplementary Table 1. The included chronic diseases.

Asthma or bronchial dilatation/chronic bronchitis
Hypertension
Heart failure
Ischemic heart disease
Diabetes
Thyroid disease (either hypothyroidism or hyperthyroidism)
Celiac disease
Inflammatory bowel disease
Psoriasis
Epilepsy
Stroke or other neurological disease
Mental health problem
Substance use problem
Sleep apnea
Obesity
